# Invasive Bladder Cancer With Peritoneal Invasion and Rectal Involvement Causing Rectal, Bilateral Ureteral, and Common Bile Duct Obstruction Without a Retroperitoneal Mass: A Case Report

**DOI:** 10.1002/iju5.70157

**Published:** 2026-02-25

**Authors:** Kazuto Imai, Norihiko Masuda, Tatsuya Hazama, Toshihide Hosomi, Keita Hanada, Takakazu Matsushita, Toshiya Akao

**Affiliations:** ^1^ Department of Urology Rakuwakai Otowa Hospital Kyoto Japan; ^2^ Department of Surgery Rakuwakai Otowa Hospital Kyoto Japan

**Keywords:** bladder cancer, malignant obstruction, pembrolizumab, retroperitoneal extension

## Abstract

**Introduction:**

Bladder cancer (BC) is rarely associated with common bile duct (CBD) obstruction. We report a case of BC with peritoneal invasion and rectal involvement complicated by bilateral ureteral and biliary obstruction.

**Case Presentation:**

A 77‐year‐old man presented with abdominal pain. Imaging revealed a bladder tumor with suspected muscle invasion, rectal obstruction, and bilateral hydronephrosis. Urothelial carcinoma was confirmed after transurethral resection of the bladder. Following the operation, the patient developed cholangitis with CBD obstruction, and in the absence of direct invasion or mass‐like lesions suggestive of malignant retroperitoneal fibrosis, retroperitoneal extension was considered a possible mechanism. Safety concerns regarding enfortumab vedotin in the setting of biliary obstruction led us to administer pembrolizumab monotherapy.

**Conclusion:**

BC may contribute to biliary obstruction through retroperitoneal processes, underscoring the need for careful diagnostic and therapeutic assessment. Furthermore, biliary drainage enabled the initiation of systemic therapy, highlighting the importance of appropriate supportive intervention.

## Keynote Message

We report a case of bladder cancer with peritoneal invasion and rectal involvement complicated by bilateral ureteral and biliary obstruction.

**Abbreviations**
BC: bladder cancer
CA125: carbohydrate antigen 125
CA19‐9: carbohydrate antigen 19‐9
CBD: common bile duct
CT: computed tomography
CTCAE: Common Terminology Criteria for Adverse Events
ECOG: Eastern Cooperative Oncology Group
EV: enfortumab vedotin
EVP: enfortumab vedotin and pembrolizumab
MMAE: monomethyl auristatin E
PS: performance status
TUR‐BT: transurethral resection of the bladder tumor
UC: urothelial carcinoma
γ‐GTP: gamma‐glutamyl transpeptidase

## 1 Introduction

Bladder cancer (BC) typically progresses via direct invasion or lymphatic and hematogenous spread and has rarely been reported in association with common bile duct (CBD) obstruction. We report a case of BC with peritoneal invasion and rectal involvement complicated by bilateral ureteral and biliary obstruction.

## 2 Case Presentation

**FIGURE 1:**
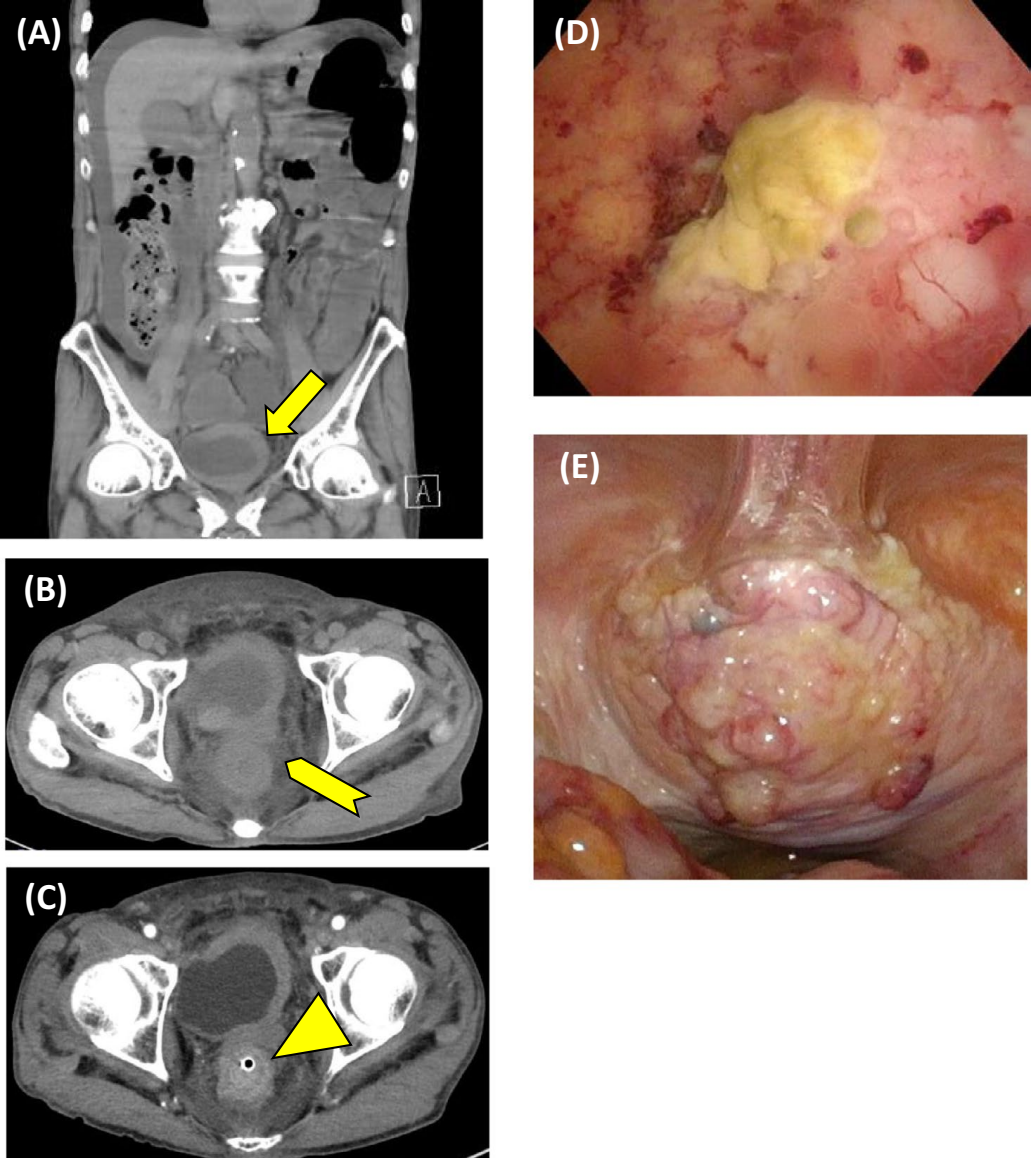
Abdominal CT revealed a bladder tumor extending from the dome to the left lateral wall, with suspected muscle invasion and left hydronephrosis (A, arrow), along with increased perirectal fat density, rectal wall thickening causing obstruction, and ascites (B, arrowhead). Contrast‐enhanced CT on hospital day 3 revealed the rectal wall alternating hypo‐ and hyperenhancement (C, triangle). Cystoscopy showed a partially necrotic mass predominantly involving the bladder dome (D). Tumor invasion extending to the peritoneum was apparent (E).

**FIGURE 2:**
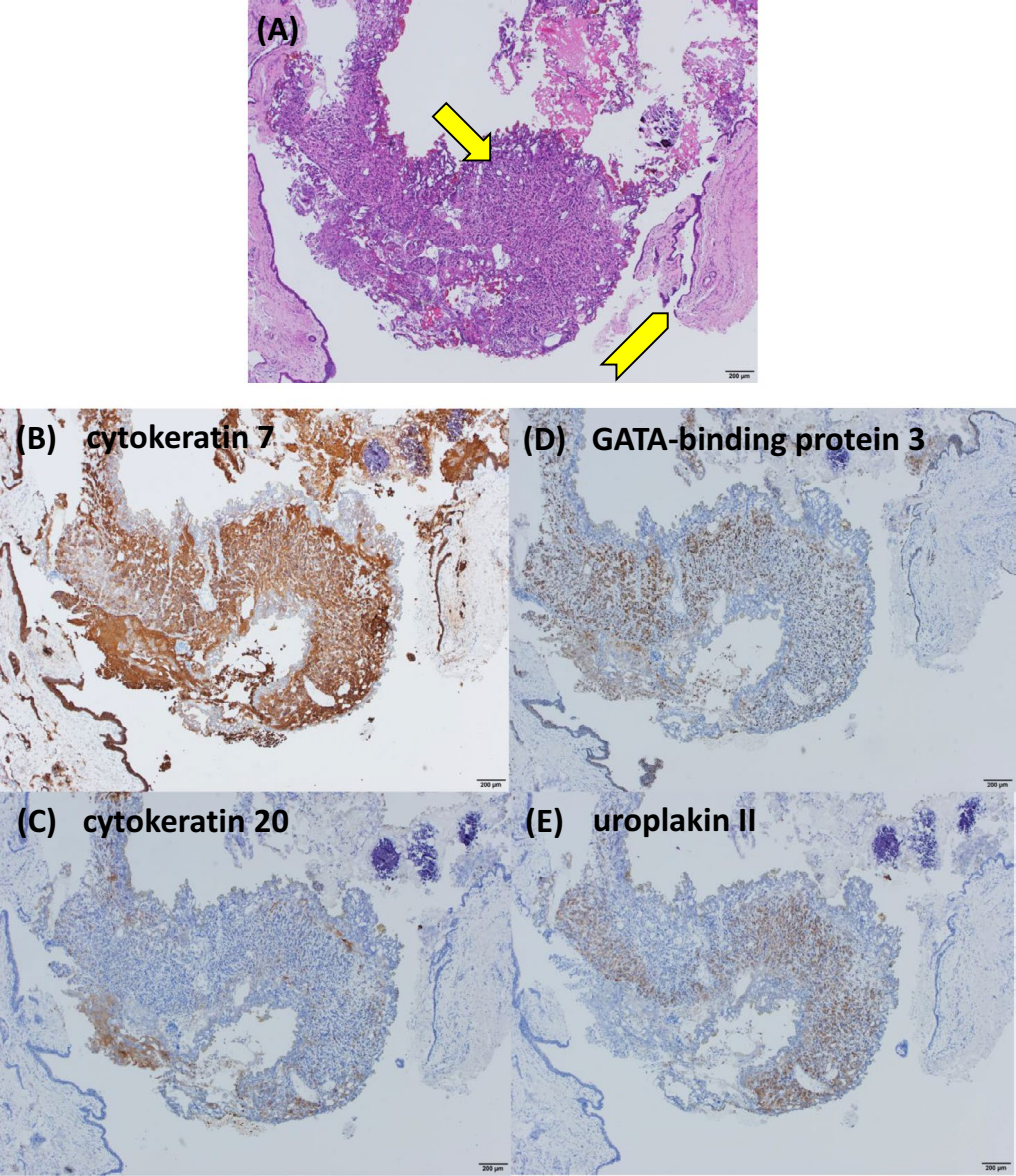
Histopathological examination of the TUR‐BT specimen revealed solid and isolated invasive proliferation (arrow) on a background of urothelium (arrowhead), with no variant histology identified (A). Immunohistochemical staining was positive for cytokeratin 7, cytokeratin 20, GATA‐binding protein 3, and uroplakin II, sequentially in that order (B–E).

**FIGURE 3:**
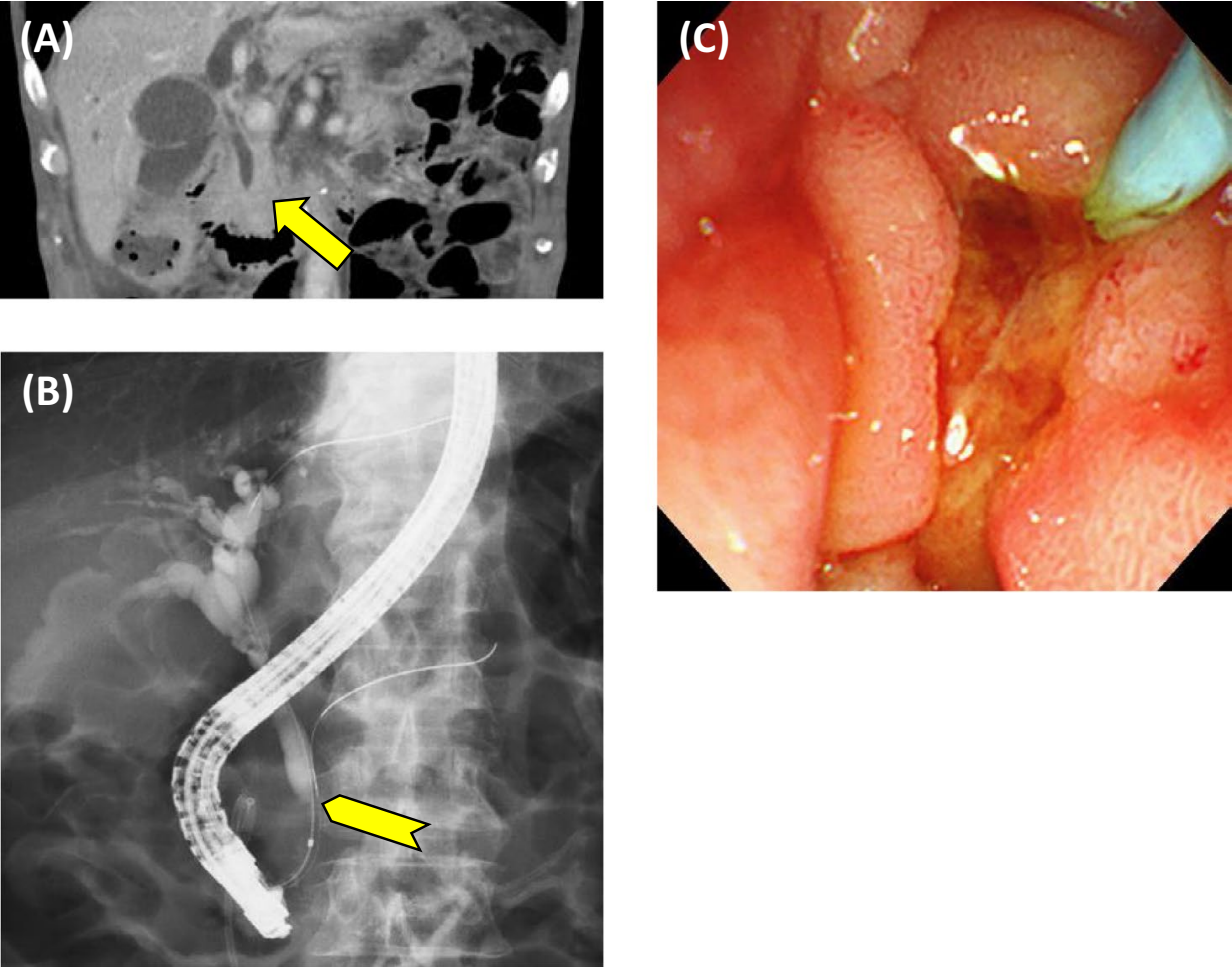
Contrast‐enhanced imaging revealed obstructive cholangitis secondary to CBD obstruction, with no evidence of direct invasion or mass‐like lesions indicative of malignant retroperitoneal fibrosis (A, arrow). Endoscopic retrograde cholangiopancreatography demonstrated beak‐like narrowing of the bile duct, suggestive of external compression (B, arrowhead), along with poor duodenal dilatation and edematous mucosa of the major duodenal papilla (C).

A 77‐year‐old man presented with abdominal pain. Abdominal computed tomography (CT) revealed a bladder tumor extending from the dome to the left lateral wall, with suspected muscle invasion and left hydronephrosis (Figure 1A), along with increased perirectal fat density, rectal wall thickening causing obstruction, and ascites (Figure 1B). No evidence of lymph node metastasis was observed. Because of the rectal obstruction, the patient was admitted and underwent colonoscopy followed by transanal decompression tube placement. Colonoscopy showed edematous mucosa at the site of rectal obstruction, and biopsy showed carcinoma with unclear mucosal architecture and differentiation, precluding identification of the primary site. Contrast‐enhanced CT on hospital day 3 revealed the development of right hydronephrosis, as well as rectal wall alternating hypo‐ and hyperenhancement, showing a malignant target sign (Figure 1C) [1]. Cystoscopy showed a patent right ureteral orifice and a partially necrotic mass predominantly involving the bladder dome (Figure 1D). Voided urine cytology was positive for malignancy. Serum carbohydrate antigen 125 (CA125), carbohydrate antigen 19–9 (CA19‐9), and Span‐1 levels were markedly elevated, whereas immunoglobulin G and immunoglobulin G4 levels were within normal range. Upper gastrointestinal endoscopy and abdominal ultrasonography revealed no evidence of gastric or pancreatic cancer. Urachal carcinoma, primary bladder adenocarcinoma, and urothelial carcinoma (UC) with glandular differentiation were considered in differential diagnosis. Transurethral resection of the bladder tumor (TUR‐BT), right ureteral stent placement, and laparoscopic sigmoid colostomy were performed on hospital day 6. Tumor invasion extending to the peritoneum was apparent (Figure 1E). No disseminated nodules were observed. Histopathological examination of the TUR‐BT specimen revealed solid and isolated invasive proliferation on a background of urothelium, with no variant histology identified (Figure 2A). No cystic components or glandular structures suggestive of urachal carcinoma or primary bladder adenocarcinoma were identified. Immunohistochemical staining showed positivity for cytokeratin 7, cytokeratin 20, GATA‐binding protein 3, and uroplakin II and negativity for caudal type homeobox 2, consistent with UC (Figure 2B–E). Cytology of ascites fluid and the rectal biopsy specimen demonstrated similar immunostaining patterns. Based on these findings, the patient was diagnosed with BC with peritoneal invasion, accompanied by malignant ascites and rectal involvement. Following the operation, the patient exhibited increased hepatobiliary enzyme levels, including aspartate aminotransferase, alanine aminotransferase, and gamma‐glutamyl transpeptidase (γ‐GTP), reaching a maximum of Common Terminology Criteria for Adverse Events (CTCAE) Grade 4. Contrast‐enhanced CT revealed obstructive cholangitis secondary to CBD obstruction, with no evidence of direct invasion or mass‐like lesions indicative of malignant retroperitoneal fibrosis. (Figure 3A). Endoscopic retrograde cholangiopancreatography demonstrated beak‐like narrowing of the bile duct, suggestive of external compression (Figure 3B), along with poor duodenal dilatation and edematous mucosa of the major duodenal papilla (Figure 3C). No endoscopic findings suggested cholangiocarcinoma, and bile duct cytology was negative. After biliary stenting, hepatobiliary enzyme abnormalities improved overall, with only γ‐GTP remaining mildly elevated at CTCAE Grade 2. The patient's Eastern Cooperative Oncology Group (ECOG) performance status (PS) was 1 at the initial presentation; however, it progressively deteriorated due to cancer progression and did not improve after biliary stenting. On postoperative day 44, at the time of initiation of systemic therapy, the ECOG PS had declined to 3, and the estimated glomerular filtration rate calculated using the Cockcroft–Gault formula was 24.4 mL/min. Based on these considerations, pembrolizumab monotherapy was initiated. Systemic therapy was ineffective, and the patient died of cancer on postoperative day 57.

## 3 Discussion

BC typically progresses via direct invasion or lymphatic and hematogenous spread, commonly affecting the lymph nodes, lungs, and peritoneum [2, 3]. Progression along the cavity or membranous structures surrounding the bladder, rectum, or retroperitoneum without formation of a discrete mass is rare [4]. In our case, the left ureter was obstructed, likely owing to direct invasion. Although imaging did not demonstrate obvious involvement of the rectum, right ureter, or CBD, histology confirmed UC in the rectum, and extrinsic tumor compression was suspected in the right ureter and CBD. On contrast‐enhanced CT, bladder and rectal wall thickening with alternating rectal wall enhancement suggests malignant rectal involvement [1]. In our patient, malignant retroperitoneal fibrosis as the cause of CBD obstruction could not be excluded; however, no typical retroperitoneal mass was observed. Alternatively, biliary obstruction may have resulted from retroperitoneal extension of BC along the retromesenteric plane, which surrounds the CBD and extends to the pelvis [5]. In this case, endoscopic drainage was performed for biliary obstruction. However, although malignant ascites was present, there was no definitive evidence of peritoneal dissemination; therefore, drainage by surgical bypass may also be considered depending on the clinical context.

Tumor markers associated with adenocarcinoma, such as CA125 and CA19‐9, are frequently elevated in urachal carcinoma [6]. In our case, histopathological and immunohistochemical findings supported the diagnosis of UC [7]; however, because radical cystectomy was not performed and the evaluation was limited to TUR‐BT specimens, the possibility that urachal carcinoma or adenocarcinoma components were not detected cannot be excluded.

Both the 2025 European Association of Urology and National Comprehensive Cancer Network guidelines recommend enfortumab vedotin plus pembrolizumab (EVP) as first‐line therapy for unresectable UC [8, 9]. In our case, although the patient was considered platinum‐unfit due to impaired renal function and an ECOG PS of 3, the KEYNOTE‐A39 trial exclusion criteria were not met; therefore, EVP was initially considered [10]. Enfortumab vedotin (EV) is a Nectin‐4–targeting antibody–drug conjugate containing monomethyl auristatin E (MMAE). MMAE is metabolized in the liver and is excreted via the bile [11, 12]. A case of Stevens–Johnson syndrome after EV treatment in a patient with liver cirrhosis has been reported, and patients with mild hepatic impairment show moderately increased exposure to free MMAE; however, a clear association between hepatic dysfunction and the effects of EV has not been established [13, 14]. In our patient, safety concerns regarding EV in the setting of biliary obstruction led us to administer pembrolizumab monotherapy.

This case demonstrates a rare progression pattern of UC, emphasizing the need to consider atypical modes of tumor spread in advanced disease. Furthermore, biliary drainage enabled the initiation of systemic therapy, highlighting the importance of appropriate supportive intervention in such complex cases.

## Consent

The authors have nothing to report.

## Conflicts of Interest

The authors declare no conflicts of interest.

## Data Availability

The data that support the findings of this study are available on request from the corresponding author. The data are not publicly available due to privacy or ethical restrictions.
